# Preventing Postpartum Venous Thromboembolism in 2022: A Narrative Review

**DOI:** 10.3389/fcvm.2022.886416

**Published:** 2022-04-12

**Authors:** Marc Blondon, Leslie Skeith

**Affiliations:** ^1^Division of Angiology and Hemostasis, Geneva University Hospitals and Faculty of Medicine, Geneva, Switzerland; ^2^Division of Hematology and Hematological Malignancies, Department of Medicine, University of Calgary, Calgary, AB, Canada

**Keywords:** postpartum, thrombosis, pulmonary embolism, prevention, heparin, preferences

## Abstract

The postpartum period represents the most critical time for pregnancy-associated venous thromboembolism (VTE), which is responsible for substantial morbidity and an important cause of maternal mortality. The estimated risk of postpartum VTE of about 1/1,000 deliveries can be modulated with the knowledge of maternal and obstetrical risk factors, although a precise estimate remains challenging in individuals. The use of postpartum low-dose low-molecular-weight heparins are tailored at intermediate and high-risk groups to reduce the thrombotic burden, despite the lack of dedicated randomized controlled trials. In this review, we will highlight the contemporary evidence on the risk of postpartum VTE, its stratification and its prevention. We will also discuss our knowledge on the values and preferences of women for postpartum thromboprophylaxis and their adherence to treatment.

## Introduction

Pregnancy-associated venous thromboembolism (PA-VTE) is responsible for about 10% of VTE in women (1). Activation of the coagulation system, endothelial trauma and venous stasis all contribute to the increased risk during pregnancy. Endogenous hormones induce a hypercoagulability characterized by increased levels of coagulation factors (fibrinogen, factors VII, VIII, X and von Willebrand factor), decreased levels of antithrombotic factors (protein S, protein C), an acquired resistance to the inhibition by protein C, and a decrease in fibrinolytic acitivty (decreased tissue plasminogen activator activity and an increase in plasminogen activator inhibitors 1 and 2) (1). Blood stasis is mediated through venous dilation and compression of the iliac veins (2), especially the left common iliac vein. Finally, vascular damage arises from delivery.

The clinical relevance of PA-VTE is underlined by its mortality and morbidity. Approximately 1/100'000 pregnant women dies from pulmonary embolism in the Western World (3). Compared to the non-pregnant setting, deep vein thromboses (DVT) are more commonly proximal with involvement of the iliofemoral veins. Postpartum DVT is complicated by a high rate of post-thrombotic syndrome, which reduces long-term quality of life (4, 5). Further, because direct oral anticoagulants are contra-indicated during breastfeeding, there is the necessity to use low-molecular-weight heparins or vitamin K antagonists in the postpartum, each with their associated constraints.

The biggest potential impact of PA-VTE prevention (thromboprophylaxis) is in the postpartum period, defined as the 6 weeks after delivery, with the peak incidence of PA-VTE in the first 2 weeks after delivery (6). Compared with non-pregnant women, women in the postpartum period have up to a 22–60-fold increased risk of VTE (7, 8). However, because the absolute VTE risk remains low in the short-term, identification of individuals who may benefit from preventive measures is needed but can be complex, such as through risk stratification. In this narrative review, we will summarize the current knowledge on risk factors and risk stratification for postpartum VTE, on our knowledge of the benefit and risk of mechanical and pharmacological thromboprophylaxis, and on patients' preferences and values relating to postpartum thromboprophylaxis. These topics will be put in the perspective of a hypothetical postpartum situation.

### Case Presentation

A 35-year old woman has delivered today her first newborn of 3,600 grams, at 39 weeks of gestation. Because of fetal distress during labor, an emergency cesarean section (C-section) was performed. The mother has no prior history or family history of VTE, and apart from obesity (BMI 32.7 based on pre-pregnancy weight 89 kg and height 165 cm) has no other medical conditions. Both mother and newborn are doing well a few hours after delivery. The mother asks about the need for prevention of venous thromboembolism.

## Overall Risk of Postpartum VTE

The absolute risk of VTE in the 6 weeks postpartum is low, based on population-based studies from several countries. A major strength of population-based studies is their large sample size and generalizability, but the definition of VTE commonly relies on administrative codes and/or some signal of anticoagulation use, without adjudication for VTE, so there is a potential for misclassification. In the UK, >200,000 pregnancies without a prior history of VTE were identified in a network of 255 general practices between 1987 and 2004, with a risk of postpartum VTE of 0.5/1,000 deliveries (8). In Denmark, a nationwide prospective cohort studied >900,000 pregnancies between 1995 and 2009, and after exclusion of women with a prior VTE, the risk was 0.35/1,000 deliveries (9). In California, between 2005 and 2010, among >1,600,000 pregnancies, the risk was 2.8/1,000 deliveries (10). In Canada, among >3,800,000 pregnancies, the risk was 1.2/1,000 deliveries between 1991 and 2006 (11). Finally, a cohort of Medicaid and private insurances in the United States between 2005 and 2011 found a risk of 1.6/1,000 deliveries (12). Underestimation of risks is likely in the first 2 studies (0.35–0.5/1,000 deliveries) due to exclusion of women with prior VTE and an unknown sensitivity of the algorithm of identification of VTE outcomes. Overestimation is possible in the 2 last studies (1.2–2.8/1,000 deliveries) due to broad diagnostic codes with suboptimal positive predictive value. Overall, these studies suggest that about 1/1,000 women will experience a VTE in the postpartum period, with a proportion of 40% of patients experiencing pulmonary embolism (6). This means that women without any VTE risk factors and women combining several risk factors will have a risk lower and >0.1%, respectively.

A 0.1% risk of postpartum VTE is 10–50 times lower than that of medical inpatients deemed at high risk of VTE (1–5%) with an indication for thromboprophylaxis (13). It is also >10 times lower than the VTE incidence after hip or knee replacement therapy (14). Although the actual number of postpartum VTE events is large because of a huge denominator of >10 million deliveries per year in Europe and North America, universal postpartum thromboprophylaxis cannot be advised for this uncommon event: in an optimistic scenario of a 70% relative reduction of VTE by short-term low-molecular-weight heparin (LMWH), one should need to treat about 1,500 women to prevent 1 VTE event. This number needed to prevent is likely too high from the perspective of healthcare costs and likely women themselves. The key is to stratify women at different risk levels, to avoid treating women at very low risk and to reduce the thrombotic risk in women at high risk, to find the optimal balance of reducing VTE while minimizing cost and possible side effects of pharmacologic thromboprophylaxis.

## Risk Stratification

There is surprisingly little direct data to quantify the absolute postpartum VTE risk among patients with additional transient or pregnancy-specific risk factors. Most of the available data used to support clinical practice guidelines has been derived from large population-based registries or case-control studies. While not an exhaustive list, we highlight the type and level of data available when trying to predict postpartum VTE risk.

### Previous VTE

Undoubtedly, a history of any prior VTE represents the most important risk factor, with a relative risk >20–50 and an absolute risk of postpartum VTE of 6–8% without thromboprophylaxis (15, 16).

### Cesarean Delivery

In a comprehensive meta-analysis that evaluated both case-control and cohort studies published up to 2015, the postpartum VTE risk after cesarean delivery was increased >3 times, compared with vaginal deliveries (17). The absolute risk from prospective studies was 2.6–4.3/1,000 deliveries, or about 1 in 230–380 deliveries. This risk was greater in urgent/emergency cesarean deliveries than planned/elective cesarean deliveries. Significant heterogeneity was observed in the meta-analysis, reflecting not only differences in research methodology but also in clinical contexts and the occurrence of other risk factors.

### Elevated BMI

There is a positive gradual association between postpartum VTE risk and BMI. In a hospital-based case control study that compared women with objectively verified VTE during pregnancy or postpartum vs. controls, the risk of postpartum VTE was modestly higher among women with a BMI ≥ 25 kg/m^2^ at the beginning of pregnancy [adjusted Odds Ratio (aOR)2.4, 95% CI, 1.7–3.3] (18). Other studies have looked at different pre-pregnancy BMI cut-offs found that compared to a normal BMI, categories of increasing BMI had progressively increased VTE risk, with class III obesity (BMI ≥40 kg/m^2^) having the highest risk (aOR 4.0, 95% CI, 2.7–6.3) (19). Excess weight gain during pregnancy has been less studied, and whether it is a risk factor for postpartum VTE or not is inconsistent (18, 19). Due to its increasing prevalence and its strength of association with VTE, obesity carries an important population attributable risk for postpartum VTE.

### Markers of Placental Disease

Intrauterine growth restriction (IUGR), pre-term birth and pre-eclampsia are well-recognized risk factors for postpartum VTE. In a population-based case-control study, postpartum women who delivered neonates with low birth weight (<2,500 grams) had a 3-fold increased risk of VTE that persisted after adjusting for possible confounding variables (aOR 2.98, 95% CI 1.80–4.93) (20). In other studies, IUGR, preterm birth (defined as <37 weeks), and pre-eclampsia showed similar VTE risk (18, 21, 22). How preeclampsia or IUGR is defined, including what growth restriction reference standard or what percentile cut-offs are used, remains unclear and may change across countries.

### Additional VTE Risk Factors

Many other VTE factors exist, with minor or intermediate associations with postpartum VTE, such as postpartum hemorrhage, infection, current or recent smoking, or medical conditions including diabetes. While bedrest during pregnancy is a known VTE risk factor, indications for strict bedrest are now uncommon (18). Also, the relationship of thrombophilia and family history with VTE is complex and goes beyond the scope of this review, but has been recently meta-analyzed (23) and detailed in guidelines (24).

Two areas of uncertainty are worth discussing. First, the timing of postpartum VTE may vary according to the type of VTE risk factors. In a UK database study, those with preterm birth or postpartum hemorrhage had increased VTE incidence rate only in the first 3 weeks postpartum. In comparison, those with an elevated BMI ≥30 kg/m^2^ or those having cesarean delivery had a risk that persisted up to 6 weeks postpartum (22). Given the multiple risk factors to evaluate and the numbers of patients and VTE cases needed, little information is still known about the timing of postpartum VTE events for different risk factors. Second, the impact of combined risk factors needs to be clarified, especially because almost half of women carry multiple risk factors in the puerperium (25). For example, when a patient has an elevated BMI ≥ 25 kg/m^2^ and with strict antepartum immobilization, the aOR for postpartum VTE may be as high as 40-times, compared with a patient who has normal BMI and no antepartum immobilization.

Currently, guidelines suggest to risk stratify using empiric schemes of levels or combination of risk factors in several categories: no thromboprophylaxis or mechanical thromboprophylaxis only, short-term pharmacologic thromboprophylaxis (days) and 6-weeks of pharmacologic thromboprophylaxis. Importantly, such guidelines (ACOG (26), RCOG (27), ASH (24)), which are detailed elsewhere, diverge dramatically in the proportion of women with advised thromboprophylaxis, between 7 and 40% for all deliveries (28) and 0.2–73% for cesarean deliveries (29). Logically, a higher prevalence of use of thromboprophylaxis is associated with lower risks among those with thromboprophylaxis, and greater numbers needed to treat to prevent 1 VTE.

A recent innovation in this field is the development of a risk score for postpartum VTE (“Maternity Clot Risk”), combining in complex forms the following 11 maternal and obstetrical factors: age, BMI, varicose veins, co-morbidities, smoking, pre-eclampsia, bleeding, infection, delivery method, parity and infant birth weight (30). The score allows estimation of postpartum VTE risk in individual women with VTE risk factors, and so may help focus prevention efforts on women above a certain threshold of risk. This score does not apply to women with a prior VTE or take into account thrombophilia. It was externally validated using a Swedish database and a UK primary care database (31), however with some limitations (32). Further validation effort would be welcome, and meanwhile it has not been incorporated into clinical practice guidelines yet.

### Case Discussion

The patient has two intermediate risk factors for VTE: obesity (BMI = 32.7 kg/m^2^) with a relative risk of 2.5 (19), and emergency C-section with a relative risk of about 4. Assuming a baseline risk among women without any risk factors of 0.05%, we could broadly estimate, with a combination of risk factors between 5.5 (additive model) or 10 (multiplicate model), that her personal postpartum VTE risk is around 0.3–0.5%. The use of the Maternity Clot Risk calculator yields a lower estimate of risk of 0.1%. We inform the patient that her risk of postpartum VTE lies around 0.1–0.5%, or about 1 in 200–1,000 deliveries.

## How Can We Prevent Postpartum VTE in High-Risk Situations?

Strong evidence shows that low-dose heparins, either unfractionated heparin or LMWH, reduces the risk of DVT and pulmonary embolism in medical or surgical inpatients, by about 50–70% (33). In the obstetric setting, the level of evidence is close to null, and has been recently summarized in an updated Cochrane systematic review as having a “very uncertain effect” (34). Indeed, all available randomized trials included small sample sizes, and some were only pilot randomized trials to test feasibility. No conclusion can be drawn with regards to the efficacy and safety of heparins in this population. Further, most concluded that the feasibility of a large-scale randomized trial was poor due to barriers and low recruitment of postpartum participants (Table 1).

**Table 1:** Published (pseudo)-randomized trials of heparins vs. placebo or no treatment to prevent postpartum venous thromboembolism.

	**Study type**	**Country**	**Population**	**Intervention**	**Control**	**Risk of VTE (intervention / control)**	**Risk of bleeding^**a**^ (intervention / control)**
Segal et al. (35)	Monocentric RCT	Israel	210 postpartum women with varicose veins	UFH 5000 IU s.c. (4–5d)	No treatment	0.8/5.3%^b^	Not reported
Hill et al. (36)	Pilot RCT	UK	50 women after elective CS	UFH 1000 IU b.i.d. (5d)	Saline b.i.d. (5d)	0/0%	4/8%
Burrows et al. (37)	Pilot RCT	Australia	76 women after CS	Dalteparin 2500 IU o.d. (5d)	Placebo o.d. (5 d)	2.6/0%	0/0%
Gates et al. (38)	Multicenter pilot RCT	UK	141 women after CS	Enoxaparin 40 mg o.d. (max 14d)	Placebo o.d. (max 14d)	1.5/0%	0/0%
Rodger et al. (39)	Multicenter pilot RCT	USA-Canada	25 women at intermediate risk	Dalteparin 5000 IU o.d. (3w)	Placebo o.d. (3w)	0/0%	0/0%
Rodger et al. (40)	Multicenter pilot RCT	USA-Canada	37 women at intermediate risk	Dalteparin 5000 IU o.d. (10d)	No treatment	0/0%	6.3/0%
Alalaf SK et al. (41)	Monocentric sequential clinical trial	Iraq	7,020 women at intermediate risk based on RCOG guidelines	Bemiparin 3500 IU o.d. / enoxaparin 40 mg o.d. (6d)	No treatment	0.1/0.4%	Not reported

*a Various definitions according to individual studies*.

*b Clinical diagnoses of VTE*.

*RCT, randomized controlled trial; UK, United Kingdom; CS, cesarean section; UFH, unfractionated heparin; d, days; w, weeks*.

Although non-randomized, a large monocentric trial in Iraq sequentially allocated to 6 days of low-dose bemiparin or enoxaparin or no treatment among women after vaginal or cesarean deliveries who were deemed at intermediate risk of postpartum VTE according to the RCOG guidelines (Table 1) (41). Quite surprisingly, the investigators reported the inclusion of 7,020 participants, with an extremely low 0.5% refusal to participate, and a 0% loss to follow-up. Enoxaparin and bemiparin were associated with a 89–95% relative reduction in the risk of symptomatic VTE, which was not adjudicated. This corresponded to a 0.3% absolute risk reduction (number needed to treat of 333). This study brings hope that short-term LMWH may efficiently reduce postpartum VTE, but no firm interpretations should be made based on its limitations of methodology, the unknown external validity and the lack of reports on safety (bleeding complications).

We have limited evidence from observational studies. Recently, two monocentric studies from the USA did not show a reduction in VTE events after implementation of standardized postpartum LMWH protocols for postpartum women with VTE risk factors, despite an increase in the use of postpartum enoxaparin from <1– >30% among 9,766 deliveries (42) and from 1 to 16% among 24,299 deliveries (43). Importantly, there was also an increased risk of wound hematomas and unplanned procedures noted in the post-LMWH protocol implementation group in one of the studies (43). Limitations to these studies include a retrospective study design evaluating a pre- and post- intervention over time that is not randomized, and the lack of VTE and bleeding event independent adjudication. These contradictory studies highlight the uncertainty still present and need for more research in the area.

Another drug of interest is low-dose aspirin. Its main advantages are its oral route and known safety profile including with breastfeeding, with a demonstrated benefit of VTE in other settings [surgical thromboprophylaxis (14), secondary prevention of VTE (44)], although with a potential lower VTE risk reduction than that of LMWH. Aspirin is currently not recommended in the postpartum period but is the subject of an ongoing trial (pilot PARTUM trial, described below).

Direct oral anticoagulants should be avoided in breastfeeding women due to safety, and there is currently no data for VTE prevention in postpartum non-breastfeeding women. With previous safety signals reported for increased heavy menstrual bleeding for women taking direct oral anticoagulants for VTE management, further research is still needed on the safety of this approach in postpartum non-breastfeeding women.

Mechanical thromboprophylaxis, in particular intermittent pneumatic compression (IPC), may also reduce the risk of VTE after surgery (45). Unfortunately, as there are no clinical data to evaluate IPC in the postpartum period, the role of mechanical thromboprophylaxis in this setting is unclear. Also, one study pointed out a low adherence with compression stockings, after hospital discharge, highlighting its burden despite its safety (46).

### Case Discussion

With the use of short-term LMWH (up to 10 days), we believe that the risk of postpartum VTE of our patient may be halved, however, the true benefits of LMWH are still unknown. In other words, 400–2,000 women would have to be treated to prevent 1 VTE event. We communicate these estimates to the patient, including the large uncertainty, possible LMWH side effects, and the suggestion by some guidelines (but not all) to prescribe LMWH in her situation. We also acknowledge that each of the authors has a different approach, including a variation in the duration of LMWH ranging from the hospital stay only (47), up to 10 days postpartum (27).

## What do Women Think of Postpartum Pharmacological Thromboprophylaxis?

Views and opinions of patients are critical in this area of current uncertainty. Strikingly, we know very little on the preferences and values of women about thromboprophylactic strategies and which threshold of VTE risk they believe should justify the use of short-term postpartum LMWH, but this could be critically helpful.

Patient preferences and values for decision-making about antepartum thromboprophylaxis have been explored in an international multicenter study (40, 48). Using a series of different exercises (direct choice, utilities for health states and probability trade-off), the authors interviewed 123 women with a history of VTE who were pregnant or considering pregnancy. There were only ~80% of women who would consider taking antepartum LMWH for a VTE risk of 10%, which is above a threshold that most physicians would recommend thromboprophylaxis. This highlights the contrast between the vision of VTE specialists and that of women with a history of VTE. While this study does not apply to a primary thromboprophylaxis decision (women without a history of VTE) in the postpartum period, it underlines the importance of shared decision-making about VTE risk.

To our knowledge, and quite surprisingly, the value and preferences of women toward postpartum thromboprophylaxis or postpartum VTE research have not been explored. We are unaware of the preferred threshold of VTE risk that would justify the use of postpartum LMWH according to pregnant women, how decisions are made that take into account the burden and side effects of LMWH, and how these views' may differ from their healthcare providers. Additionally, how patients' views should be incorporated into clinical practice guidelines is largely unknown.

Today, a large difference of VTE risks to justify postpartum LMWH exists between experts' opinion (formulating guidelines) and the actual practice from these guidelines. When using the Maternity Clot Risk to indirectly estimate VTE risk used in guidelines, in a sample of parturients from the Geneva University Hospitals (28), we found that the 2015 RCOG and the 2018 ACOG guidelines suggested thromboprophylaxis at a risk of 0.12 and 0.20%, respectively. This contrasts dramatically with experts, who advocate for a VTE risk of 1–3% to justify postpartum VTE (24). Clearly, more research is needed in this field, to better appreciate women's preferences and values, and ensure that the use of postpartum LMWH achieves an acceptable number needed to prevent a VTE event, while minimizing harm.

Also of interest is the adherence to postpartum thromboprophylaxis, which may be suboptimal. In a 2018 study completed in Israel at a tertiary center, 250 postpartum women completed a telephone interview at the end of their planned postpartum thromboprophylaxis (48). While in-hospital adherence with LMWH was 100% in-hospital, 33% had injected <80% of the planned mean 7 days of LMWH after discharge, and 18% had injected none. Women were more likely to be compliant if they had used LMWH in the past or antenatally, and women who felt they had received good technical explanations about injections were more likely to be compliant. The two main reasons for non-adherence were the belief that LMWH was not necessary and challenges with injections at home. These reasons were similar to the reasons described for non-participation in the pilot feasibility randomized trial PROSPER, that evaluated the role of 10–21 days of post-discharge LMWH vs. no LMWH for women with intermediate VTE risk factors (40).

Three prospective studies from the UK suggest more optimistic estimates of adherence (46). Among 51 women who completed a prospective diary of postpartum LMWH injections for a duration of 7 days to 6 weeks, 82% had not missed more than most 1 dose. Among 95 women who had an indication for both antenatal and postnatal LMWH, mostly at a prophylactic dose, 98% in the antepartum and 93% in the postpartum had an adherence ≥80% (49). Similarly, another prospective cohort from the UK indicate an 83% proportion of complete adherence with postpartum LMWH (50). The selection of highly motivated women willing to participate in clinical studies in the two first studies and the overall prospective design likely boosted the level of adherence. Whether such a high adherence is representative of the general population is doubtful. Together, these studies highlight the importance of discussing the benefit of LMWH and the technique of LMWH injections prior to discharge for those who benefit.

### Case Resolution

The patient has understood her risks, possible benefits of LMWH and the overall uncertainty to guide the decision of thromboprophylaxis. While she would have met inclusion criteria for the pilot PARTUM trial (described below), this study was not available at her center. After shared decision making, she decides to use low-dose LMWH for 10 days to further reduce her risks of VTE. She receives training on subcutaneous injections.

## Discussion

Throughout this review, we have tried to highlight several areas in critical need for high-quality data.

With regards to risk stratification, the advent of an estimator of individual risks (the Maternity Clot Risk) may be of great help to clinicians in the future, but needs, in our opinion, further validation, ideally in a population of women with intermediate VTE risk factors who have not received postpartum thromboprophylaxis. Individual-patient data meta-analyses of observational studies may help increase the power to detect clinically significant interactions of common risk factors. The identification of the mode of combination (additive, multiplicative, supra-additive) requires large sample sizes, which may emanate from individual patient analysis meta-analyses. Lastly, further research is needed to better understand patient experience and associated preferences and values, to better guide research and clinical practice guidelines in the area of postpartum thromboprophylaxis.

It is a clear paradox that, only in the UK, close to 300,000 women receive postpartum thromboprophylaxis every year, but that pilot randomized trial of subcutaneous LMWH concluded on the unfeasibility of a large-scale trial of 10,000–20,000 women. The COVID-19 pandemic has brought into light the challenges of recruitment, but also the possibility of international collaborations that can bring answers to clinically important questions through large randomized trials, and we should not stop our effort for postpartum VTE. The pilot PARTUM trial, an ongoing pilot randomized trial testing the feasibility of conducting a large trial of low-dose aspirin vs. placebo for postpartum women at intermediate risk of VTE, led by one of the authors, is a great example of such a global effort (https://partumtrial.ca; clinicaltrials.gov ID: NCT04153760). Randomized trials of low-dose LMWH vs. no treatment are still desperately needed to provide high-quality data to support current thromboprophylaxis practice patterns for postpartum women at intermediate risk of VTE. Not only will such randomized trials allow to draw conclusions on the efficacy of these different drugs, but also on their safety and the actual risks of VTE in control groups.

## Author Contributions

MB and LS drafted the review, revised it critically, and provided approval for publication of its content.

## Conflict of Interest

The authors declare that the research was conducted in the absence of any commercial or financial relationships that could be construed as a potential conflict of interest.

## Publisher's Note

All claims expressed in this article are solely those of the authors and do not necessarily represent those of their affiliated organizations, or those of the publisher, the editors and the reviewers. Any product that may be evaluated in this article, or claim that may be made by its manufacturer, is not guaranteed or endorsed by the publisher.
